# Combining glycosylated hemoglobin A_1c_ and fasting plasma glucose for diagnosis of type 2 diabetes in Chinese adults

**DOI:** 10.1186/1472-6823-13-44

**Published:** 2013-10-08

**Authors:** Miao Mo, Weijian Zhong, Genming Zhao, Ye Ruan, Hua Zhang, Liang Shi, Dajiang Lu, Qundi Yang, Yanyun Li, Qingwu Jiang, Rui Li, Wang-Hong Xu

**Affiliations:** 1 Department of Epidemiology, School of Public Health; Key Laboratory of Public Health Safety, Ministry of Education, Fudan University, 138 Yi Xue Yuan Road, Shanghai 200032, People’s Republic of China; 2 Shanghai Municipal Center for Disease Control and Prevention, 1380 Zhong Shan Xi Road, Shanghai 200336, People’s Republic of China; 3 School of Sports Science, Shanghai University of Sport, 399 Chang Hai Road, Shanghai 200438, People’s Republic of China

**Keywords:** Type 2 diabetes, Diagnosis, Glycosylated hemoglobin A_1c_, Fasting plasma glucose, Chinese adults

## Abstract

**Background:**

Glycosylated hemoglobin A_1c_ (HbA_1c_) has been applied to identify type 2 diabetes (T2DM) in the U.S. and European countries. It has not been used in China mainly due to lack of a standardized approach to measure HbA_1c_, short of knowledge about racial-specific standard and deficiency of an optimal cut-off point.

**Methods:**

To evaluate combination of HbA_1c_ and fasting plasma glucose (FPG) in diagnosing T2DM in Chinese adults, a multistage sampling cross-sectional study was conducted in Shanghai, China, in 2009. The FPG measurement, HbA_1c_ assay, and oral glucose tolerance test (OGTT) were performed in 6,661 Chinese adults (3057 men, 3604 women) who had no prior history of diabetes to identify the unrecognized T2DM.

**Results:**

A total of 454 participants were identified as T2DM based on the 1999 World Health Organization (WHO) diagnostic criteria. Of these patients, 239 were detected using an FPG ≥ 7.0 mmol/l and 141 were further identified using an HbA_1c_ ≥ 43 mmol/mol (6.1%), achieving a sensitivity of 83.7% and a specificity of 89.3% for combining use of FPG and HbA_1c_. In subjects at high risk of diabetes, the combining use of FPG and HbA_1c_ produced a higher sensitivity and an improved positive predictive value (PPV), and had a satisfactory specificity and negative predictive value (NPV).

**Conclusions:**

The combining use of FPG and HbA_1c_ is a potential screening and diagnosis approach for T2DM in Chinese adults, especially among those at high risk of the disease.

## Background

Type 2 diabetes mellitus (T2DM) is a common disease reflecting metabolic disorders characterized with hyperglycemia, which may lead to specific long-term complications affecting heart, brain, eyes, kidneys and nervous system [1]. Currently, diagnosis of T2DM in Chinese adults is principally according to the 1999 World Health Organization (WHO) diagnostic criteria [2], namely, a person can be diagnosed as T2DM when he or she has 1) a random plasma glucose ≥ 11.1 mmol/l accompanied by typical symptoms of diabetes such as thirst and polyuria; or 2) a fasting plasma glucose (FPG) ≥ 7.0 mmol/l; or 3) a 2-hour plasma glucose (2hPG) after an oral glucose tolerance test (OGTT) ≥ 11.1 mmol/l [3]. These diagnostic criteria are determined based on the relationship between glucose levels and the risk of subsequent long-term complications.

The accuracy of glucose measurement, however, is more or less affected by the pre-analytic instability and biologic variability of glucose concentrations within and between days. Moreover, OGTT requires a second blood sample and is therefore more costly and time-consuming, bringing inconvenient in logistics and causing uncomfortable feeling in individuals. Compared with 2hPG in OGTT, FPG measurement is more reproducible and simpler to perform in clinics and communities [4]. Therefore FPG measurement is usually used alone to screen T2DM in China [5]. Due to that a certain proportion of T2DM patients have a normal FPG but an elevated 2hPG (≥ 11.1 mmol/l) [6], almost half diabetes patients remain undiagnosed in China [7]. A more efficient and practical method is needed to identify the individuals with diabetes in general population.

Hemoglobin A_1c_ (HbA_1c_), a glycosylated form of hemoglobin, reflects one’s plasma glucose levels over past 2–3 months and is less influenced by recent diet and emotional stress [8]. HbA_1c_ level is a convenient and reliable measurement of chronic glycemia [9], and can be tested concurrently with FPG at fasting. HbA_1c_ is associated with the risk of long-term diabetic complications and has been used to monitor glycemic control status in prevalent patients with diabetes for about 30 years. Substantial studies [10-12], including one conducted in Chinese population [12], have proved that HbA_1c_ may be a helpful tool for diagnosing T2DM. The American Diabetes Association (ADA) has used an HbA_1c_ cut-point of 48 mmol/mol (6.5%) as one of diagnostic criteria for T2DM in its 2010 Clinical Practice Recommendations [1].

Although HbA_1c_ has been applied to identify T2DM in the U.S. and European countries, it has not been used in China, mainly due to lack of a standardized approach to measure HbA_1c_, short of knowledge about racial-specific standard and deficiency of an optimal cut-point for detecting T2DM in the population. HbA_1c_ values between 37 mmol/mol (5.5%) and 48 mmol/mol (6.5%) have been associated with a substantially increased risk for developing T2DM [13]. Due to racial disparity in HbA_1c_ level [14], however, the optimal threshold varies across populations. In Chinese adults at high risk of glucose intolerance, combining use of an FPG ≥ 6.1 mmol/l and an HbA_1c_ ≥ 43 mmol/mol (6.1%) has been shown to predict subsequent T2DM accurately [15]. Bao *et al.*[12] reported that an HbA_1c_ ≥ 45 mmol/mol (6.3%) can be used as a diagnostic criterion for diabetes in Chinese adults when FPG and OGTT are not available. Given that the HbA_1c_ value reflects average level of glycemia over the preceding 2–3 months and the FPG represents the current glucose concentration, combining use of the two indicators may improve the accuracy of diagnosis.

In this study, we included 6,661 Chinese adults with no prior history of diabetes to evaluate whether the combination of FPG and HbA_1c_ can be used as a tool for screening and diagnosis of T2DM in Chinese community settings.

## Methods

### Study design and population

**Figure 1:**
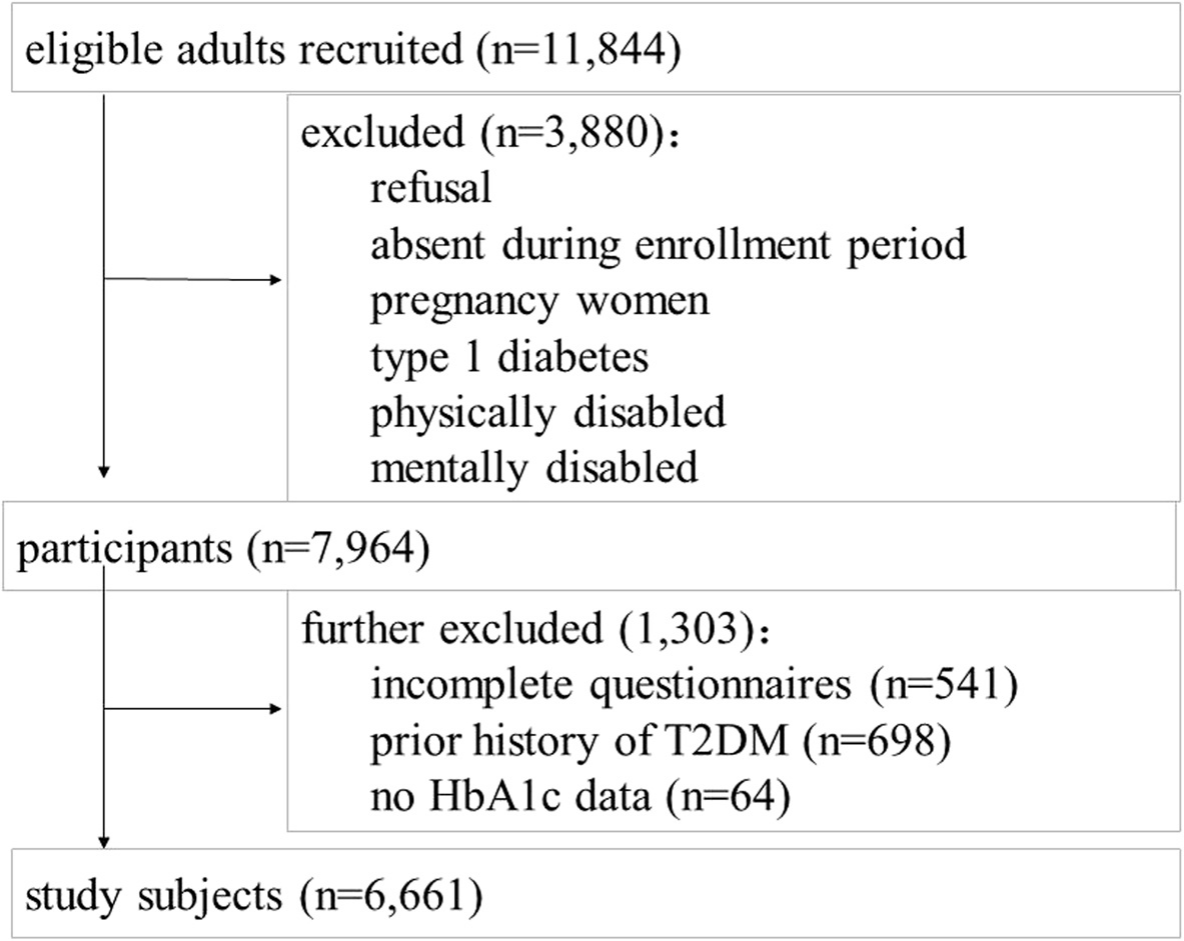
Flow diagram of recruitment of participants.

A cross-sectional survey for T2DM was conducted in Chinese adults in Shanghai, China, in 2009. As described in our previous report [7], a multistage sampling process was applied to select a representative sample of the residents of Shanghai who were at ages of 35–74 years old. Briefly, 4 districts and 2 counties were randomly selected from all 12 districts and 7 counties. Then 1–2 sub-districts or towns were randomly selected from each selected district or county. And then, 1–2 communities or villages were randomly selected from each selected sub-district or town. Finally, 1,000-2,000 eligible subjects (permanent residents of Shanghai, 35–74 years old and having been in the city for at least 5 years) were randomly selected from each selected community or village and invited to participate in the survey. Among 11,844 eligible adults recruited, 7,964 participated in the survey, resulting in a response rate of 67.2%. The main reasons for no-response were refusal to participate and absent during the period of enrollment. Pregnant women, individuals with type 1 diabetes, and those physically or mentally disabled were also excluded from participation. After further excluding subjects with incomplete questionnaires and those having a prior history of T2DM or missing values of HbA_1c_, we finally included 6,661 subjects (3,057 men and 3,604 women) in our analysis (Figure 1).

The Institutional Review Board at Shanghai Municipal Center of Disease Control and Prevention approved the study. Informed written consent was obtained from each participant before collecting data and bio-specimen.

### Data collection

Information on demographic characteristics and lifestyle factors of the participants was collected by trained interviewers. At the interview, body weight, standing height, waist circumstance (WC) and blood pressure was measured for each participant according to a standard protocol, as described previously [7]. Two measurements were taken and the mean value was used in the analyses. Body mass index (BMI) was calculated as weight in kilograms divided by the square of height in meters (kg/m^2^) using direct measurements. Hypertension was defined as the systolic/diastolic blood pressure (SBP/DBP) ≥ 140/90 mmHg or using antihypertensive medications due to hypertension.

### Laboratory measurements

All participants were asked to maintain their usual physical activities and diets for at least 3 days before having an OGTT. After at least 10 hours of overnight fasting, a 1–1.5 ml venous blood sample was collected for each subject in a vacuum tube containing sodium fluoride for measuring FPG, and a 3–3.5 ml non-anticoagulated venous blood sample was drawn for measuring total cholesterol (TC), triglycerides (TG), high and low density lipoprotein cholesterol (HDL-C and LDL-C). For those with an FPG < 7.0 mmol/l, a standard 75-g glucose load was given and a second blood sample was drawn at 120 minutes after the glucose load to measure 2hPG.

Biochemical assay was conducted in several Community Healthcare Centers according to a standardized protocol. Plasma glucose was tested using glucose oxidase-peroxidase (GOD-PAP) method and serum cholesterol and triglyceride levels were assayed enzymatically using commercial reagents. HbA_1c_ level was assayed using high-performance liquid chromatography (HPLC), which is recommended by the National Glycohemoglobin Standardization Program [16]. The interassay coefficient of variation (CV) was < 1.82% for FPG (SD < 0.23 mmol/l), < 1.38% for TG (SD < 0.02 mmol/l), < 1.54% for TC (SD < 0.08 mmol/l), < 1.60% for HDL-C (SD < 0.01 mmol/l), < 5.30% for LDL-C (SD < 0.21 mmol/l), and < 6.13% for HbA_1c_ (SD < 0.77). An elevated WC was defined as WC ≥ 90 cm for men or ≥ 80 cm for women, and an elevated TG level referred to a fasting serum TG level of ≥ 1.695 mmol/l. Hypertriglyceridemic waist (HW) phenotype was defined as having both an elevated WC and an elevated TG level [17].

### Statistical analysis

We used SAS version 9.1 for all statistical analyses. Medians and interquartile ranges of demographic and clinical characteristics of 6,661 participants were presented and compared by sex using Wilcoxon tests. Pearson partial correlation analysis was used to calculate the correlation coefficient of HbA_1c_ level with concentrates of FPG and 2hPG. Restricted cubic splines (RCS) were used to evaluate the potential nonlinear relationship of HbA_1c_ with FPG or 2hPG using the 5th, 25th, 75th and 95th percentiles as fixed knots. ANOVA tests and Spearman rank correlation analyses were applied to compare the differences and the trend of glycemic levels among subgroups with specific demographic and clinical characteristics. We considered *P* < 0.05 as statistically significant for a two-sided test.

The 1999 WHO diagnostic criteria for T2DM were used as the gold standard. The sensitivity was calculated as the number of subjects correctly classified as diabetes by a certain diagnosing threshold divided by the total number of diabetes by the gold standard, and specificity as the ratio of true negatives to all negatives. The Youden Index (sensitivity + specificity – 1) was used to identify the optimal cut-off point based on ROC curve [18]. Positive predictive value (PPV) was defined as the number of true positives divided by the total number of people who test positive, while negative predictive value (NPV) referred to the proportion of subjects with a negative test result who were correctly diagnosed.

## Results

Table 1 shows the demographic and clinical characteristics of participants of the survey. Compared with the men, the women had a lower average level of BMI, WC, SBP, DBP or TG but a higher concentration of TC, HDL-C, LDL-C or 2hPG (all *P* values < 0.05). No significant difference was observed for age and average levels of FPG and HbA_1c_ between men and women.

**Table 1:** Demographic and clinical characteristics of the participants of the study

**Characteristics**	**Median (25th, 75th percentile)**			***P value***
	**All subjects (n = 6,661)**	**Men (n = 3,057)**	**Women (n = 3,604)**	
Age (years)	54 (48, 61)	54 (48, 61)	54 (48, 60)	*0.6403*
BMI (kg/m^2^)	24.0 (21.9, 26.3)	24.2 (22.1, 26.3)	23.9 (21.8, 26.3)	*0.0315*
WC (cm)	82 (76, 89)	85 (79, 90)	80 (74, 87)	*<0.0001*
SBP (mmHg)	123 (113, 135)	125 (115, 137)	121 (111, 134)	*<0.0001*
DBP (mmHg)	79 (72, 85)	80 (73, 87)	79 (71, 83)	*<0.0001*
TC (mmol/l)	4.60 (4.00, 5.25)	4.45 (3.87, 5.03)	4.80 (4.14, 5.41)	*<0.0001*
TG (mmol/l)	1.35 (0.91, 2.01)	1.40 (0.92, 2.10)	1.31 (0.90, 1.95)	*0.0003*
HDL-C (mmol/l)	1.30(1.10, 1.55)	1.21 (1.03, 1.45)	1.37 (1.17, 1.60)	*<0.0001*
LDL-C (mmol/l)	2.67 (2.20, 3.09)	2.60 (2.18, 3.00)	2.71 (2.29, 3.17)	*<0.0001*
FPG (mmol/l)	5.0 (4.6, 5.4)	5.0 (4.6, 5.4)	5.0 (4.7, 5.4)	*0.0486*
2hPG (mmol/l)^a^	6.0 (5.0, 7.1)	5.9 (4.8, 7.1)	6.0 (5.1, 7.1)	*<0.0001*
HbA_1c_ (mmol/mol &%)	37 (32, 40) & 5.5 (5.1, 5.8)	37 (32, 41) & 5.5 (5.1, 5.9)	37 (33, 40) & 5.5 (5.2, 5.8)	*0.4235*

^a^For 6,422 participants who had an OGTT (2,919 men and 3,503 women). All *P* values were from Wilcoxon rank sum tests.

Presented in Additional file 1: Table S1 are the average levels of FPG, 2hPG and HbA_1c_ by demographic and clinical characteristics of the subjects. In both sexes, the levels of FPG, 2hPG and HbA_1c_ increased with age and increasing BMI, with Spearman correlation coefficients varying from 0.10 to 0.25 (all *P* < 0.05). The participants with elevated WC, hypertension, elevated TG, reduced HDL-C or HW phenotype had a higher concentration of FPG, 2hPG or HbA_1c_ than those without (*P* < 0.05), but with the difference in the FPG level between subjects with and without reduced HDL-C reaching significant only among women.

**Figure 2:**
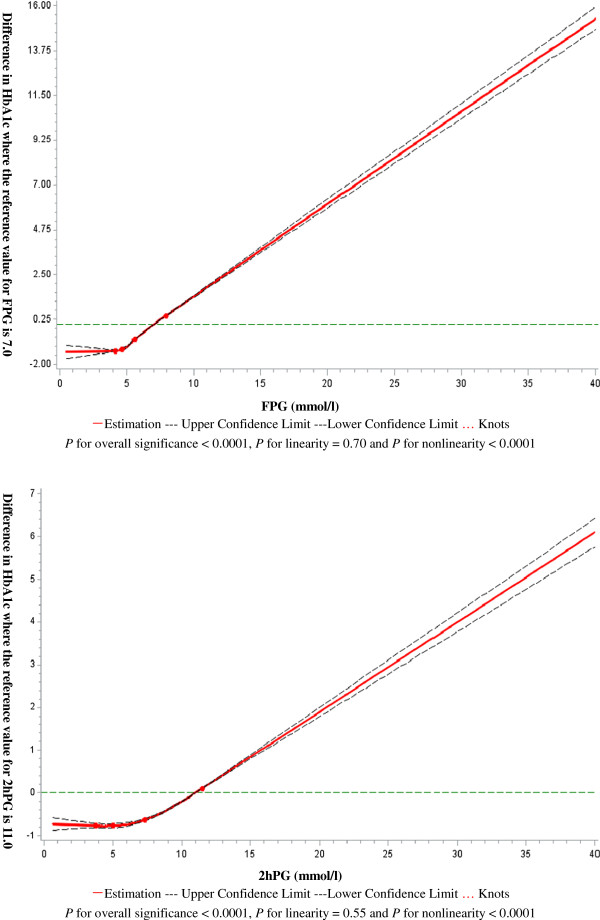
**Nonlinear relationship of HbA**_**1c **_**with FPG and 2hPG using RCS with 4 knots.**

In this population, HbA_1c_ was closely correlated with FPG (r = 0.613, *P* < 0.0001) and 2hPG (r = 0.299, *P* < 0.0001) after adjusting for age and sex. As shown in Figure 2, however, nonlinear does-response relationship was observed for HbA_1c_ level with the levels of FPG and 2hPG after accounting for age and sex (*P* values for non-linear association < 0.0001).

Using the 1999 WHO diagnostic criteria as the gold standard, the sensitivity of FPG ≥ 7.0 mmol/l in diagnosing T2DM was 52.6%, while the sensitivity and specificity of HbA_1c_ of 43 mmol/mol (6.1%), the optimal cut-point of the index in this population, reached 75.6% and 89.3%, respectively (Table 2). The optimal threshold of HbA_1c_ for detecting T2DM ranged from 40 to 45 mmol/mol (5.8-6.3%) in the subgroups stratified by sex, age, BMI, WC, TG, HDL-C or presence of hypertension or HW phenotype. These cut-points increased with age and appeared higher in subjects with an elevated TG level or HW phenotype. All the optimal cut-points performed better in sensitivity than did the FPG ≥ 7.0 mmol/l. It is of note that the PPV of the optimal threshold increased with age and increasing BMI, and was higher in the subjects with elevated WC, hypertension, elevated TG, reduced HDL-C or HW phenotype (Table 2).

**Table 2:** **Performance of the optimal HbA**_**1c **_**cut-points in participants by demographic and clinical characteristics**

**Characteristics**	**Sensitivity of FPG** ≥ **7.0 mmol/l (%)**	**Optimal HbA**_**1c **_**mmol/mol (%)**	**Performance of optimal HbA**_**1c **_**value (%)**			
			**Sensitivity**	**Specificity**	**PPV**	**NPV**
All subjects	52.6	43 (6.1)	75.6	89.3	34.1	98.0
Gender						
Male	59.0	43 (6.1)	78.2	88.8	36.7	98.0
Female	45.9	43 (6.1)	72.7	89.8	31.6	98.1
Age						
35-44	68.8	40 (5.8)	84.4	84.9	15.1	99.4
45-54	59.9	41 (5.9)	85.9	82.5	23.5	98.9
55-64	51.1	42 (6.0)	82.2	81.3	27.3	98.2
65-74	40.0	45 (6.3)	70.0	91.9	51.1	96.2
BMI						
≤23.0	52.9	43 (6.1)	73.6	92.7	27.0	99.0
23.1-24.9	58.0	43 (6.1)	75.0	90.1	32.9	98.2
≥25.0	50.6	43 (6.1)	76.4	85.3	37.8	96.9
Elevated WC^a^						
No	56.6	43 (6.1)	78.9	91.8	30.2	99.0
Yes	50.4	43 (6.1)	73.6	85.8	37.1	96.6
Hypertension^b^						
No	52.6	43 (6.1)	76.3	90.6	29.7	98.7
Yes	52.7	43 (6.1)	74.6	84.5	42.1	95.7
Elevated TG^c^						
No	50.8	41 (5.9)	83.2	83.1	18.3	99.1
Yes	53.9	43 (6.1)	78.1	85.1	39.9	96.8
Reduced HDL-C^d^						
No	56.7	43 (6.1)	72.7	89.6	31.6	98.0
Yes	46.4	43 (6.1)	79.9	88.7	38.4	98.0
HW phenotype^e^						
No	53.2	43 (6.1)	75.3	90.8	30.0	98.6
Yes	51.9	43 (6.1)	75.9	82.9	42.3	95.4

^a^WC ≥ 90 cm in men and ≥ 80 cm in women;

^b^SBP/DBP ≥ 140/90 mmHg or being on antihypertensive medications;

^c^TG ≥ 1.695 mmol/l;

^d^HDL-C < 1.036 mmol/l (men) and < 1.295 mmol/l (women);

^e^hypertriglyceridemic waist phenotype, defined as having both a high WC ( ≥ 90 cm for men, ≥ 80 cm for women) and an elevated TG level ( ≥ 1.695 mmol/l).

As shown in Additional file 2: Table S2, 239 T2DM were identified by elevated FPG (≥ 7.0 mmol/l) and 215 by increased 2hPG (≥ 11.0 mmol/l). Of the two groups of diabetes patients, 74.1% (177 of 239) and 45.6% (98 of 215) were identified as patients using an HbA_1c_ cut-point of 48 mmol/mol (6.5%) recommended by ADA, producing a sensitivity of 60.6% and a specificity of 96.2%. By using an HbA_1c_ of 45 mmol/mol (6.3%) recommended by Bao *et al.* for Chinese adults [12], the sensitivity increased to 67.2% and the specificity decreased to 94.0%. HbA_1c_ of 43 mmol/mol (6.1%) achieved a sensitivity of 75.6%, a specificity of 89.3%, a PPV of 34.1% and an NPV of 98.0% in diagnosis of T2DM.

As presented in Table 3, combining use of an FPG ≥ 7.0 mmol/l and an HbA_1c_ ≥ 43 mmol/mol (6.1%) had a higher sensitivity (83.7%) and PPV (36.4%) than using either one of the indicators alone in identify T2DM, together with a specificity of 89.3% and an NPV of 98.0%. Combining use of FPG and HbA_1c_ performed much better among the subjects with an elevated TG level or HW phenotype in the sensitivity (87.4% and 86.6% versus 83.7%) and PPV (42.7% and 44.9% versus 36.4%) than in all subjects, but with a certain decrease in the specificity (85.1% and 82.9% versus 89.3%) and NPV (98.2% and 97.4% versus 98.7%). Combining use of the two indicators was observed to classify 26.9% (272/1011) of pre-diabetes defined by 1999 WHO criteria as diabetes (Additional file 3: Table S3).

**Table 3:** Performance of FPG, 2hPG and HbA1c alone and combining use of FPG (mmol/l) and HbA1c (mmol/mol) in diagnosing diabetes

**Population**	**Subjects**	**Diabetes**	**Se (%)**	**Sp (%)**	**PPV**	**NPV**
Individual test						
By FPG alone^a^	6,661	239	52.6	100.0	100.0	96.7
By 2hPG alone^b^	6,422	215	100.0	100.0	100.0	100.0
By HbA1c alone^c^	6,661	343	75.6	89.3	34.1	98.0
Combining use of FPG and HbA_1c_						
All subjects	6,661	380	83.7	89.3	36.4	98.0
Subjects with elevated WC^d^	2,810	239	83.0	85.8	39.8	97.8
Subjects with elevated TG^e^	2,385	269	87.4	85.1	42.7	98.2
Subjects with HW^f^	1,320	162	86.6	82.9	45.5	97.4

^a^FPG ≥ 7.0 mmol/l; ^b^2 hPG ≥ 11.1 mmol/l; ^c^HbA_1c_ ≥ 43 mmol/mol (6.1%); ^d^WC ≥ 90 cm in men, ≥ 80 cm in women; ^e^TG ≥ 1.695 mmol/l; ^f^hypertriglyceridemic waist phenotype, defined as having both a high WC (≥ 90 cm for men, ≥ 80 cm for women) and an elevated TG level (≥ 1.695 mmol/l).

## Discussion

In this study including 6,661 Chinese adults randomly selected from a community setting, we find that an HbA_1c_ threshold of 43 mmol/mol (6.1%) performs best in diagnosing T2DM when combining with an FPG ≥ 7.0 mmol/l, particularly among those at a high risk of T2DM. Our results provide additional evidence on the value of HbA_1c_ measurement in identifying diabetes patients in Chinese population.

For diabetes patients, it is diabetic complications occurred subsequently that damage their health and quality of life. The value of OGTT in diagnosing diabetes lies in the close relationship of 2hPG with the risk of diabetic complications. Elevated HbA_1c_ level has also been linked to a higher risk of diabetic retinopathy [19], nephropathy [20], cardiovascular diseases [21,22] and premature death [23,24]. Moreover, HbA_1c_ possesses much lower intra-individual CV (3.6%) than 2hPG (16.7%) and FPG (5.7%), and has been used as the most reproducible and repeatable measurement of glycemic status [25]. Therefore, HbA_1c_ has great potential as a screening or diagnosing tool for T2DM.

Several cross-sectional [11,12] and prospective [10,26] studies have provided evidence on the superior performance of HbA_1c_ to FPG in screening or diagnosing T2DM. Consistent with these reports, we find that the optimal HbA_1c_ threshold of 43 mmol/mol (6.1%) had a higher sensitivity (75.6%) than did the FPG ≥7.0 mmol/l (52.6%) in our population, and produced a specificity of 89.3%, a PPV of 34.1% and an NPV of 98.0%. It is of note that, in this population, the HbA_1c_ cut-point of 45 mmol/mol (6.3%) achieved a slightly higher sensitivity (67.2% vs. 62.8%) and a slightly lower specificity (94.0% vs. 96.1%) than in Bao *et al*’s report [12]. The older average age of our population may partly explain the difference.

Interestingly, when combining the cut-point of 43 mmol/mol (6.1%) for HbA_1c_ and 7.0 mmol/l for FPG, the sensitivity and the PPV increased to 83.7% and 36.4%, respectively, with the specificity and the NPV as high as by using HbA_1c_ alone. Based on these results and the fact that HbA_1c_ is more stable and convenient than OGTT in practice [12], we recommend that the combination of an HbA_1c_ ≥ 43 mmol/mol (6.1%) and an FPG ≥ 7.0 mmol/l can be used as screening and diagnostic criteria for T2DM in Chinese population. We also find that, among the subjects with an elevated TG level or with HW phenotype, the combination of an HbA_1c_ ≥ 43 mmol/mol (6.1%) and an FPG ≥ 7.0 mmol/l achieved a satisfactory sensitivity and a greatly improved PPV, implicating that the criteria are more efficient in detecting T2DM among subgroups at high risk of T2DM.

Although it has been suggested that HbA_1c_ alone has potential to be used as a diagnostic criterion for T2DM in Chinese adults [12], combining use of the measurement with FPG has two additional advantages. Firstly, genetic variants of hemoglobin (e.g. sickle cell trait, HbS trait, HbC trait, etc.) and some medical conditions that could shorten or conversely prolong the survival of erythrocytes may affect the accuracy of HbA_1c_ measurements [27]. Combining use of HbA_1c_ and FPG can reduce the misdiagnosis of T2DM caused by using HbA_1c_ alone. Secondly, as HbA_1c_ value reflects average level of blood glucose over the preceding 2–3 months, subjects having diabetes in 3 months cannot be detected by testing HbA_1c_ alone. Combining use of HbA_1c_ with FPG can compensate for this weak point.

The main strength of the study is its large sample size. However, the response rate of 67.2% arouses a concern on the representation of the sample, and the lack of characteristics information on the non-participants limits our ability to evaluate the potential selection bias. In this population, age appeared to be the most important characteristic factor linked to different optimal HbA_1c_ cut-off points (Table 2). The older average age of our sample population due to non-response of the subjects who refused to participate or were absent during the enrolment period may lead to a higher cut-off point of HbA_1c_ for diagnosis. However, the non-inclusion of these individuals is unlikely matter in the potential application of the public health tool. Additionally, the biochemical assays were conducted in several Community Healthcare Centers, which may have led to inter-lab bias in measurements of HbA_1c_. The unified protocol and strict quality control process may help to release our concerns. Moreover, although the sensitivity and specificity of combining use of HbA_1c_ and FPG were as high as 83.7% and 89.3%, respectively, false positive and negative cannot be neglected. Finally, the high cost of HbA_1c_ test may limit its widespread use in China. Compared with FPG assay, HbA_1c_ test is much more expensive. Considering that the cost for HbA_1c_ assay almost equals to that for the OGTT [12,28], however, combining use of HbA_1c_ and FPG will not lead to an extra economic burden comparing with the current application of 1999 WHO diagnostic criteria.

## Conclusions

In conclusion, a threshold of HbA_1c_ ≥ 43 mmol/mol (6.1%) in combination with an FPG ≥ 7.0 mmol/l produce a satisfactory sensitivity and specificity for diagnosis of T2DM in our population. Our findings support the combining use of FPG and HbA_1c_ as a screening and diagnosis tool of T2DM in Chinese adults, especially among those at a high risk of the disease. Further epidemiological and clinical studies are warranted to validate our results.

## Abbreviations

2hPG: 2 hour plasma glucose; ADA: American Diabetes Association; BMI: Body mass index; DBP: Diastolic blood pressure; FPG: Fasting plasma glucose; HbA1c: Glycosylated hemoglobin A_1c_; HDL-C: High density lipoprotein cholesterol; HW: Hypertriglyceridemic waist (phenotype); IFG: Impaired fasting glucose; IGT: Impaired glucose tolerance; LDL-C: Low density lipoprotein cholesterol; NFG: Normal fasting glucose; NGT: Normal glucose tolerance; OGTT: Oral glucose tolerance test; SBP: Systolic blood pressure; T2DM: Type 2 diabetes; TC: Total cholesterol; TG: Triglycerides; WC: Waist circumference; WHO: World Health Organization.

## Competing interests

The authors declare that they have no competing interests.

## Authors’ contributions

MM drafted the manuscript. RL and WHX coordinated the study and contributed to study design, statistical analysis, and revision of the manuscript. HZ, GZ and QJ contributed to revision of the manuscript. WZ, YR, LS, DL, QY and YL contributed to data acquisition. WHX and RL are the guarantors of this work and have full access to all the data in the study and take responsibility for the integrity of the data and the accuracy of the data analysis. All authors read and approved the final manuscript.

## Pre-publication history

The pre-publication history for this paper can be accessed here:

http://www.biomedcentral.com/1472-6823/13/44/prepub

## Supplementary Material

Additional file 1: Table S1 Glycemic levels (mean ± SD) in male and female subjects by demographic and clinical characteristics. Click here for file

Additional file 2: Table S2 Distribution of participants with different glycemic status stratified by WHO recommended criteria and several HbA_1c_ thresholds. Click here for file

Additional file 3: Table S3 Performance of combining use of FPG (mmol/l) and HbA_1c_ (mmol/mol) in detecting glycemic status defined by WHO criteria. Click here for file
