# Supplementary material for: Combining glycosylated hemoglobin A1c and fasting plasma glucose for diagnosis of type 2 diabetes in Chinese adults
Source: BMC Endocr Disord. 2013 Oct 8;13:44. doi: 10.1186/1472-6823-13-44 (PMC3853138; doi:10.1186/1472-6823-13-44)
Supplement: Additional file 1: Table S1 — Glycemic levels (mean ± SD) in male and female subjects by demographic and clinical characteristics. [file 1472-6823-13-44-S1.doc]

**Additional file 1: Table S1. Glycemic levels (mean ± SD) in male and female subjects by demographic and clinical characteristics**

| **Characteristics** | **Men** | | | | |  | **Women** | | | | |
| --- | --- | --- | --- | --- | --- | --- | --- | --- | --- | --- | --- |
|  | **No. of subjects** | **FPG (mmol/l)** | **2hPG (mmol/l)** | **HbA_1c_**  **(mmol/mol, %)** | |  | **No. of subjects** | **FPG (mmol/l)** | **2hPG (mmol/l)** | **HbA_1c_**  **(mmol/mol, %)** | |
| All subjects | 3,057 | 5.2 ± 1.2 | 6.2 ± 2.1 | 38 ± 10 | 5.6 ± 0.9 |  | 3,604 | 5.1 ± 1.0 | 6.3 ± 1.9 | 38 ± 9 | 5.6 ± 0.8 |
| Age |  |  |  |  | |  |  |  |  |  | |
| 35-44 | 511 | 4.9 ± 1.2 | 5.5 ± 1.5 | 36 ± 9 | 5.4 ± 0.8 |  | 525 | 4.9 ± 0.7 | 5.7 ± 1.5 | 34 ± 8 | 5.3 ± 0.7 |
| 45-54 | 1,061 | 5.2 ± 1.2 | 6.1 ± 2.1 | 38 ± 10 | 5.6 ± 0.9 |  | 1,346 | 5.1 ± 1.0 | 6.0 ± 1.8 | 37 ± 9 | 5.5 ± 0.8 |
| 55-64 | 1,030 | 5.3 ± 1.3 | 6.3 ± 2.1 | 38 ± 10 | 5.6 ± 0.9 |  | 1,258 | 5.2 ± 1.0 | 6.6 ± 2.0 | 38 ± 9 | 5.6 ± 0.8 |
| 65-74 | 455 | 5.3 ± 1.2 | 7.0 ± 2.4 | 39 ± 11 | 5.7 ± 1.0 |  | 475 | 5.3 ± 0.9 | 7.1 ± 2.2 | 39 ± 9 | 5.7 ± 0.8 |
| *P(r)* ^Ɨ^ |  | *<.0001(0.19)* | *<.0001(0.19)* | *<.0001(0.14)* | |  |  | *<.0001(0.18)* | *<.0001(0.24)* | *<.0001(0.17)* | |
| BMI |  |  |  |  | |  |  |  |  |  | |
| <23.0 | 1,070 | 5.0 ± 1.3 | 5.8 ± 2.0 | 37 ± 9 | 5.5 ± 0.8 |  | 1,394 | 5.0 ± 0.7 | 5.8 ± 1.6 | 36 ± 7 | 5.4 ± 0.6 |
| 23.1-24.9 | 776 | 5.2 ± 1.3 | 6.2 ± 2.0 | 38 ± 10 | 5.6 ± 0.9 |  | 862 | 5.2 ± 1.2 | 6.3 ± 1.8 | 38 ± 10 | 5.6 ± 0.9 |
| ≥25.0 | 1,210 | 5.3 ± 1.2 | 6.5 ± 2.2 | 39 ± 10 | 5.7 ± 0.9 |  | 1,347 | 5.3 ± 1.0 | 6.9 ± 2.2 | 39 ± 9 | 5.7 ± 0.8 |
| *P(r)* ^Ɨ^ |  | *<.0001(0.19)* | *<.0001(0.15)* | *<.0001(0.10)* | |  |  | *<.0001(0.19)* | *<.0001(0.25)* | *<.0001(0.16)* | |
| Elevated WC ^a^ |  |  |  |  | |  |  |  |  |  | |
| No | No | 2,139 | 5.1 ± 1.2 | 6.0 ± 2.0 | 37 ± 9 |  | 1,710 | 5.0 ± 0.9 | 5.9 ± 1.6 | 37 ± 8 | 5.5 ± 0.7 |
| Yes | Yes | 917 | 5.4 ± 1.3 | 6.7 ± 2.3 | 40 ± 10 |  | 1,893 | 5.2 ± 1.0 | 6.8 ± 2.1 | 38 ± 9 | 5.6 ± 0.8 |
| *P value* ^ǂ^ |  | *<.0001* | *<.0001* | *<.0001* | |  |  | *<.0001* | *<.0001* | *<.0001* | |
| Hypertension ^b^ |  |  |  |  | |  |  |  |  |  | |
| No | 2,295 | 5.1 ± 1.2 | 6.0 ± 2.0 | 38 ± 10 | 5.6 ± 0.9 |  | 2,837 | 5.0 ± 0.9 | 6.1 ± 1.8 | 37 ± 8 | 5.5 ± 0.7 |
| Yes | 762 | 5.4 ± 1.3 | 6.8 ± 2.4 | 39 ± 10 | 5.7 ± 0.9 |  | 767 | 5.5 ± 1.3 | 7.1 ± 2.3 | 40 ± 10 | 5.8 ± 0.9 |
| *P value* ^ǂ^ |  | *<.0001* | *<.0001* | *<.0001* | |  |  | *<.0001* | *<.0001* | *<.0001* | |
| Elevated TG ^c^ |  |  |  |  | |  |  |  |  |  | |
| No | 1,903 | 5.0 ± 0.9 | 6.0 ± 2.0 | 37 ± 9 | 5.5 ± 0.8 |  | 2,373 | 5.0 ± 0.9 | 6.0 ± 1.7 | 37 ± 8 | 5.5 ± 0.7 |
| Yes | 1,154 | 5.4 ± 1.6 | 6.6 ± 2.3 | 39 ± 11 | 5.7 ± 1.0 |  | 1,231 | 5.3 ± 1.1 | 7.0 ± 2.3 | 39 ± 10 | 5.7 ± 0.9 |
| *P value* ^ǂ^ |  | *<.0001* | *<.0001* | *<.0001* | |  |  | *<.0001* | *<.0001* | *<.0001* | |
| Reduced HDL-C ^d^ |  |  |  |  | |  |  |  |  |  | |
| No | 2,269 | 5.2 ± 1.2 | 6.1 ± 2.1 | 38 ± 10 | 5.6 ± 0.9 |  | 2,179 | 5.1 ± 1.0 | 6.2 ± 1.9 | 37 ± 8 | 5.5 ± 0.7 |
| Yes | 788 | 5.2 ± 1.3 | 6.4± 2.3 | 39 ± 11 | 5.7 ± 1.0 |  | 1,425 | 5.2 ± 1.0 | 6.5 ± 2.0 | 38 ± 9 | 5.6 ± 0.8 |
| *P value* ^ǂ^ |  | *0.6440* | *0.0003* | *0.0146* | |  |  | *0.0019* | *<.0001* | *0.0002* | |
| HW phenotype ^e^ |  |  |  |  | |  |  |  |  |  | |
| No | 2,566 | 5.1 ± 1.2 | 6.1 ± 2.1 | 38 ± 10 | 5.6 ± 0.9 |  | 2,774 | 5.1 ± 0.9 | 6.1 ± 1.7 | 37 ± 8 | 5.5 ± 0.7 |
| Yes | 490 | 5.5 ± 1.5 | 6.8 ± 2.4 | 40 ± 11 | 5.8 ± 1.0 |  | 830 | 5.4 ± 1.1 | 7.3 ± 2.4 | 40 ± 10 | 5.8 ± 0.9 |
| *P value* ^ǂ^ |  | *<.0001* | *<.0001* | *<.0001* | |  |  | *<.0001* | *<.0001* | *<.0001* | |

^Ɨ^ *P* values were from Spearman rank correlation tests, while *r* value referred to the Spearman correlation coefficient;

^ǂ^ *P* values were from the Analysis of Variance (ANOVA);

^a^ WC ≥ 90 cm in men and ≥ 80 cm in women;

^b^ SBP/DBP ≥ 140/90 mmHg or being on antihypertensive medications;

^c^ TG ≥ 1.695 mmol/l;

^d^ HDL-C< 1.036 mmol/l (men), < 1.295 mmol/l (women);

^e^ hypertriglyceridemic waist phenotype, defined as having both a high WC ( ≥ 90 cm for men, ≥ 80 cm for women) and an increased TG level ( ≥ 1.695 mmol/l).
