# Supplementary material for: Combining glycosylated hemoglobin A1c and fasting plasma glucose for diagnosis of type 2 diabetes in Chinese adults
Source: BMC Endocr Disord. 2013 Oct 8;13:44. doi: 10.1186/1472-6823-13-44 (PMC3853138; doi:10.1186/1472-6823-13-44)
Supplement: Additional file 2: Table S2 — Distribution of participants with different glycemic status stratified by WHO recommended criteria and several HbA1c thresholds. [file 1472-6823-13-44-S2.doc]

**Additional file 2: Table S2. Distribution of participants with different glycemic status stratified by WHO recommended criteria and several HbA_1c_ thresholds**

|  | **75 g Oral Glucose Tolerance Test** | | | | **Total** |
| --- | --- | --- | --- | --- | --- |
|  | **NGT**  **(n=5,329)** | **IGT**  **(n=878)** | **Diabetes**  **(n=215 )** | **Not assayed**  **(n=239)** |  |
| FPG, mmol/l |  |  |  |  |  |
| < 6.1 (NFG) | 5,196 (97.5) | 755 (86.0) | 119 (55.3) | 0 (0.0) | 6,070 (91.1) |
| 6.1-6.9 (IFG) | 133 (2.5) | 123 (14.0) | 96 (44.7) | 0 (0.0) | 352 (5.3) |
| ≥ 7.0 (Diabetes) | 0 (0.0) | 0 (0.0) | 0 (0.0) | 239 (100.0) | 239 (3.6) |
| HbA_1c_, mmol/mol (%) |  |  |  |  |  |
| ≥ 43 (6.1) ^a^ | 437 (8.2) | 226 (25.7) | 141 (65.6) | 202 (84.5) | 1,006 (15.1) |
| ≥ 45 (6.3) ^b^ | 227 (4.3) | 144 (16.4) | 116 (54.0) | 189 (79.1) | 676 (10.2) |
| ≥ 48 (6.5) ^c^ | 143 (2.7) | 91 (10.4) | 98 (45.6) | 177 (74.1) | 509 (7.6) |

NGT: normal glucose tolerance, 2hPG < 7.8 mmol/l;

NFG: normal fasting glucose, FPG < 6.1 mmol/l;

IGT: impaired glucose tolerance, 7.8 mmol/l ≤ 2hPG < 11.1 mmol/l;

IFG: impaired fasting glucose, 6.1 mmol/l ≤ FPG < 7.0 mmol/l;

Diabetes: FPG ≥ 7.0 mmol/l or 2hPG ≥ 11.1 mmol/l;

^a^ Optimal threshold in this population;

^b^ Threshold recommended by Bao *et al* [15];

^c^ Threshold recommended by ADA
