# Supplementary material for: Combining glycosylated hemoglobin A1c and fasting plasma glucose for diagnosis of type 2 diabetes in Chinese adults
Source: BMC Endocr Disord. 2013 Oct 8;13:44. doi: 10.1186/1472-6823-13-44 (PMC3853138; doi:10.1186/1472-6823-13-44)
Supplement: Additional file 3: Table S3 — Performance of combining use of FPG (mmol/l) and HbA1c (mmol/mol) in detecting glycemic status defined by WHO criteria. [file 1472-6823-13-44-S3.doc]

**Additional file 3: Table S3. Performance of combining use of FPG (mmol/l) and HbA_1c_ (mmol/mol) in detecting glycemic status defined by WHO criteria**

| **Glycemic status by WHO criteria** | **FPG <7.0** | **FPG <7.0** | **FPG ≥7.0** | **FPG ≥7.0** | **Total** |
| --- | --- | --- | --- | --- | --- |
|  | **HbA1c<43 (6.1%)** | **HbA1c ≥43 (6.1%)** | **HbA1c <43 (6.1%)** | **HbA1c ≥43 (6.1%)** |  |
| All subjects | 5,618 | 804 | 37 | 202 | 6,661 |
| Non-diabetes | 5,544 | 663 | 0 | 0 | 6,207 |
| NFG & NGT | 4,805 | 391 | 0 | 0 | 5,196 |
| Isolated IFG | 87 | 46 | 0 | 0 | 133 |
| Isolated IGT | 593 | 162 | 0 | 0 | 755 |
| IFG and IGT | 59 | 64 | 0 | 0 | 123 |
| Diabetes | 74 | 141 | 37 | 202 | 454 |
| by FPG only | 0 | 0 | 37 | 202 | 239 |
| by 2hPG only | 74 | 141 | 0 | 0 | 215 |
| With elevated WC ^a^ | 2,212 | 453 | 27 | 118 | 2,810 |
| Non-diabetes | 2,163 | 359 | 0 | 0 | 2,522 |
| NFG & NGT | 1,769 | 192 | 0 | 0 | 1,961 |
| Isolated IFG | 40 | 26 | 0 | 0 | 66 |
| Isolated IGT | 313 | 102 | 0 | 0 | 415 |
| IFG and IGT | 41 | 39 | 0 | 0 | 80 |
| Diabetes | 49 | 94 | 27 | 118 | 288 |
| by FPG only | 0 | 0 | 27 | 118 | 145 |
| by 2hPG only | 49 | 94 | 0 | 0 | 143 |
| With elevated TG ^b^ | 1,834 | 406 | 25 | 120 | 2,385 |
| Non-diabetes | 1,800 | 316 | 0 | 0 | 2116 |
| NFG & NGT | 1,443 | 172 | 0 | 0 | 1615 |
| Isolated IFG | 37 | 21 | 0 | 0 | 58 |
| Isolated IGT | 292 | 88 | 0 | 0 | 380 |
| IFG and IGT | 28 | 35 | 0 | 0 | 63 |
| Diabetes | 34 | 90 | 25 | 120 | 269 |
| by FPG only | 0 | 0 | 25 | 120 | 145 |
| by 2hPG only | 34 | 90 | 0 | 0 | 124 |
| With HW phenotype ^c^ | 964 | 259 | 20 | 77 | 1,320 |
| Non-diabetes | 939 | 194 | 0 | 0 | 1,133 |
| NFG & NGT | 730 | 99 | 0 | 0 | 829 |
| Isolated IFG | 18 | 14 | 0 | 0 | 32 |
| Isolated IGT | 168 | 57 | 0 | 0 | 225 |
| IFG and IGT | 23 | 24 | 0 | 0 | 47 |
| Diabetes | 25 | 65 | 20 | 77 | 187 |
| by FPG only | 0 | 0 | 20 | 77 | 97 |
| by 2hPG only | 25 | 65 | 0 | 0 | 90 |

NGT: normal glucose tolerance, 2hPG < 7.8 mmol/l;

IGT: impaired glucose tolerance, 7.8 mmol/l ≤ 2hPG < 11.1 mmol/l;

NFG: normal fasting glucose, FPG < 6.1 mmol/l;

IFG: impaired fasting glucose, 6.1 mmol/l ≤ FPG < 7.0 mmol/l;

^a^ WC ≥ 90 cm in men and ≥ 80 cm in women;

^b^ TG ≥ 1.695 mmol/l;

^c^ hypertriglyceridemic waist phenotype, defined as having both a high WC ( ≥ 90 cm for men, ≥ 80 cm for women) and an elevated TG level ( ≥ 1.695 mmol/l).
